# Supplementary material for: Sex Differences in Management, Time to Intervention, and In-Hospital Mortality of Acute Myocardial Infarction and Non-Myocardial Infarction Related Cardiogenic Shock
Source: J Clin Med. 2024 Dec 31;14(1):180. doi: 10.3390/jcm14010180 (PMC11721040; doi:10.3390/jcm14010180)
Supplement: Supplementary file 1 [file jcm-14-00180-s001.zip › jcm-3373029-supplementary.pdf]

Supplementary Figure S1. Study population selection flowchart.
Abbreviations: CS, cardiogenic shock, AMI, acute myocardial infarction.

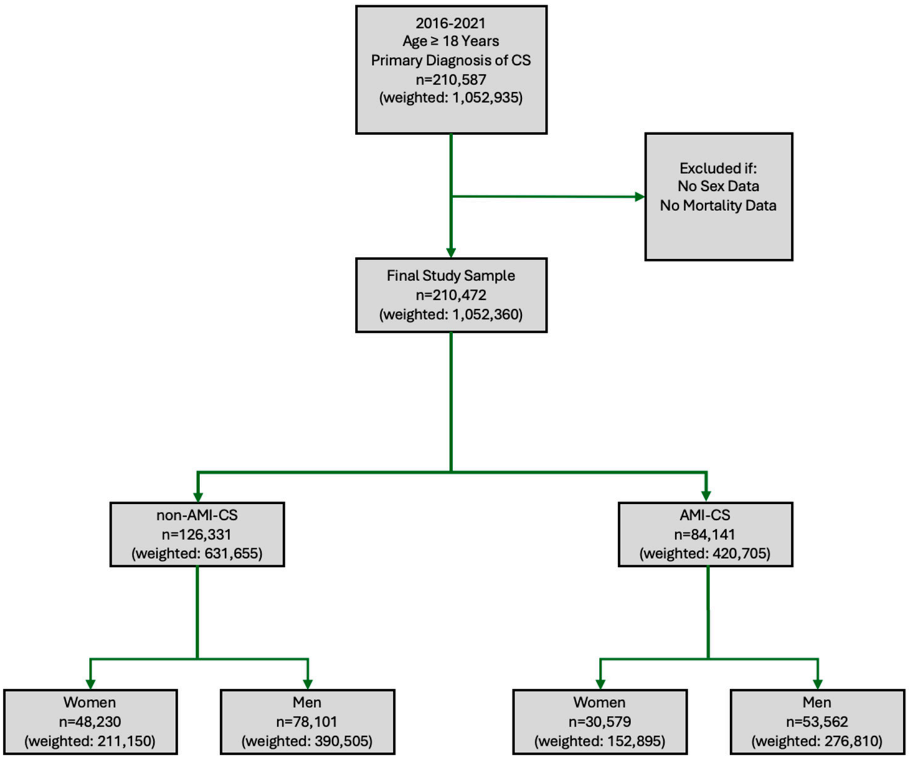

Supplementary Table S1. ICD-10 diagnosis and procedure codes used during analysis.

Diagnosis	ICD-10 Diagnosis Codes
Cardiogenic Shock	R57.0
Obesity	E66*
Dyslipidemia	E78*
Diabetes	E08*, E09*, E10*, E11*, E13*, O24.0*, O24.1*, O24.3*
Chronic Hypertension	I10*, I11*, I12*, I13*, I15*, O10*, O11*
Tobacco Use Disorder	099.33*, Z72.0, F17.21*, F17.22*, F17.29*
Peripheral Vascular Disease	I702*, I703*, I704*, I705*, I706*, I707*, I739
Congestive Heart Failure	I25.5, I42*, I43*, I50.1, I50.20, I50.22, I50.23, I50.30, I50.32, I50.33, I50.40, I50.42, I50.43, I50.810, I50.812, I50.813, I50.814, I50.82, I50.83, I50.84, I50.89, I50.9

Chronic Liver Disease	K70* K71* K72* K73* K74* K75* K76* K77*
Valvular Heart Disease	I05*, I06*, I07*, I08*, I34*, I35*, I36*, I37*
Coronary Artery Disease	I25.1*, I25.8*
Stroke	I63*
Prior Percutaneous Coronary Intervention	Z9861, Z955
Prior Coronary Artery Bypass Graft	Z951, I257, I25810, I25812 V4581, 41402, 41403, 41404, 41405, 41407
Procedure	ICD-10 Procedure Codes
Coronary Artery Bypass Graft	0210*, 0211*, 0212*, 0213*, B202*, B203*, B212*, B213*, B223*, B233*
Percutaneous Coronary Intervention	0270346, 027034Z, 0270356, 027035Z, 0270366, 027036Z, 0270376, 027037Z, 02703D6, 02703DZ, 02703E6, 02703EZ, 02703F6, 02703FZ, 02703G6, 02703GZ, 02703T6, 02703TZ, 02703Z6, 02703ZZ, 0270446, 027044Z, 0270456, 027045Z, 0270466, 027046Z, 0270476, 027047Z, 02704D6, 02704DZ, 02704E6, 02704EZ, 02704F6, 02704FZ, 02704G6, 02704GZ, 02704T6, 02704TZ, 02704Z6, 02704ZZ, 0271346, 027134Z, 0271356, 027135Z, 0271366, 027136Z, 0271376, 027137Z, 02713D6, 02713DZ, 02713E6, 02713EZ, 02713F6, 02713FZ, 02713G6, 02713GZ, 02713T6, 02713TZ, 02713Z6, 02713ZZ, 0271446, 027144Z, 0271456, 027145Z, 0271466, 027146Z, 0271476, 027147Z, 02714D6, 02714DZ, 02714E6, 02714EZ, 02714F6, 02714FZ, 02714G6, 02714GZ, 02714T6, 02714TZ, 02714Z6, 02714ZZ, 0272346, 027234Z, 0272356, 027235Z, 0272366, 027236Z, 0272376, 027237Z, 02723D6, 02723DZ, 02723E6, 02723EZ, 02723F6, 02723FZ, 02723G6, 02723GZ, 02723T6, 02723TZ, 02723Z6, 02723ZZ, 0272446, 027244Z, 0272456,027245Z, 0272466, 027246Z, 0272476, 027247Z, 02724D6, 02724DZ, 02724E6, 02724EZ, 02724F6, 02724FZ, 02724G6, 02724GZ, 02724T6, 02724TZ, 02724Z6, 02724ZZ, 0273346, 027334Z, 0273356, 027335Z, 0273366, 027336Z, 0273376, 027337Z, 02733D6, 02733DZ, 02733E6, 02733EZ, 02733F6, 02733FZ, 02733G6, 02733GZ, 02733T6, 02733TZ, 02733Z6, 02733ZZ, 0273446, 027344Z, 0273456, 027345Z, 0273466, 027346Z, 0273476, 027347Z, 02734D6, 02734DZ, 02734E6, 02734EZ, 02734F6, 02734FZ, 02734G6, 02734GZ, 02734T6, 02734TZ, 02734Z6, 02734ZZ, 02C03Z6, 02C03ZZ, 02C04Z6, 02C04ZZ, 02C13Z6, 02C13ZZ, 02C14Z6, 02C14ZZ, 02C23Z6, 02C23ZZ, 02C24Z6, 02C24ZZ, 02C33Z6, 02C33ZZ, 02C34Z6, 02C34ZZ
Intra-Aortic Balloon Pump	5A02110, 5A02210
Percutaneous Left	02HA3RZ, 02HA3RS, 02HA0RZ, 02HA3QZ, 02HA3RJ, 5A0221D, 5A0211D, 5A02116, 5A02216

Ventricular Assist Device	
Extracorporeal Membrane Oxygenation	5A1522F, 5A1522G, 5A15223, 5A1522H
Right Heart Catheterization	4A023N6, 02HP32Z, 4A1239Z, 4A133B3, 02HP30Z, 02HQ30Z, 02HQ32Z, 02HR30Z, 02HR32Z, 4A0239Z, 4A023N8, 4A03353, 4A033B3, 4A13353
Left Ventricular Assist Device	02HA0QZ
Cardiac Transplant	02YA0Z0, 02YA0Z1, 02YA0Z2

Supplementary Table S2. Odds ratios of procedural and clinical outcomes in patients with AMI-CS.

Outcomes in Women compared to Men	Unadjusted Odds Ratio	p-value	Model 1 (Only Demographics) Odds Ratio	p-value	Model 2 (All Baseline Characteristics) Odds Ratio	p-value
**Procedural Outcomes**						
Revascularization	0.72 (0.70-0.75)	<0.001	0.80 (0.78-0.83)	<0.001	0.85 (0.82-0.88)	<0.001
PCI	0.84 (0.81-0.86)	<0.001	0.91 (0.88-0.94)	<0.001	0.98 (0.95-1.01)	0.260
CABG	0.65 (0.62-0.68)	<0.001	0.69 (0.66-0.72)	<0.001	0.72 (0.69-0.75)	<0.001
MCS	0.67 (0.65-0.69)	<0.001	0.74 (0.72-0.76)	<0.001	0.76 (0.74-0.79)	<0.001
IABP	0.74 (0.71-0.76)	<0.001	0.80 (0.77-0.82)	<0.001	0.82 (0.79-0.85)	<0.001
pLVAD	0.67 (0.64-0.70)	<0.001	0.73 (0.70-0.77)	<0.001	0.76 (0.72-0.80)	<0.001
ECMO	0.58 (0.53-0.64)	<0.001	0.71 (0.64-0.79)	<0.001	0.71 (0.64-0.79)	<0.001
RHC	0.85 (0.82-0.88)	<0.001	0.90 (0.87-0.94)	<0.001	0.90 (0.88-0.96)	<0.001
Advanced Heart Failure Therapy	0.56 (0.45-0.70)	<0.001	0.71 (0.57-0.89)	0.003	0.68 (0.54-0.86)	0.001
LVAD	0.51 (0.40-0.66)	<0.001	0.63 (0.48-0.82)	0.001	0.60 (0.46-0.79)	<0.001
Heart Transplant	0.73 (0.48-1.12)	0.153	1.00 (0.64-1.58)	0.982	0.95 (0.61-1.49)	0.832
**Clinical Outcomes**						
In-hospital Mortality	1.20 (1.17-1.24)	<0.001	1.11 (1.07-1.14)	<0.001	1.10 (1.07-1.14)	<0.001
IMV	1.05 (1.02-1.08)	0.001	1.06 (1.03-1.09)	<0.001	1.06 (1.03-1.09)	<0.001
Major Bleeding	0.95 (0.91-0.98)	0.003	0.98 (0.95-1.03)	0.491	0.98 (0.94-1.02)	0.301
AKI	0.80 (0.78-0.82)	<0.001	0.75 (0.72-0.77)	<0.001	0.72 (0.70-0.74)	<0.001
AKI Requiring Dialysis	0.83 (0.78-0.88)	<0.001	0.84 (0.79-0.90)	<0.001	0.85 (0.79-0.90)	<0.001
Stroke	1.65 (1.29-2.10)	<0.001	1.55 (1.20-2.00)	0.001	1.52 (1.18-1.97)	0.001

Procedural outcomes include interventions performed at any point during hospitalization. All analyses involve multivariate logistic regression and are survey weight adjusted. Model 1 includes adjustment with year of NIS data, age group, race, household income quartile, insurance status, hospital type and teaching status. Model 2 includes adjustment with all model 1 covariates as well as past medical history of diabetes mellitus, hypertension, dyslipidemia, tobacco use, obesity, chronic kidney disease, liver disease, peripheral vascular disease, coronary artery disease, chronic heart failure, valvular heart disease, stroke, prior PCI, and prior CABG.

Abbreviations: AMI-CS, acute myocardial infarction cardiogenic shock; MCS, mechanical support device; RHC, right heart catheterization; IMV, invasive mechanical ventilation; AKI, acute kidney injury.

Supplementary Table S3. Odds ratios of procedural and clinical outcomes in patients with non-AMI-CS.

Outcomes in Women compared to Men	Unadjusted Odds Ratio	p-value	Model 1 (Only Demographics) Odds Ratio	p-value	Model 2 (All Baseline Characteristics) Odds Ratio	p-value
**Procedural Outcomes**						
Revascularization	0.60 (0.58-0.63)	<0.001	0.62 (0.58-0.60)	<0.001	0.77 (0.73-0.81)	<0.001
PCI	0.78 (0.72-0.85)	<0.001	0.79 (0.73-0.86)	<0.001	1.04 (0.96-1.14)	0.331
CABG	0.56 (0.53-0.59)	<0.001	0.59 (0.56-0.62)	<0.001	0.72 (0.68-0.76)	<0.001
MCS	0.68 (0.66-0.71)	<0.001	0.74 (0.71-0.77)	<0.001	0.76 (0.74-0.80)	<0.001
IABP	0.67 (0.64-0.71)	<0.001	0.72 (0.68-0.76)	<0.001	0.77 (0.73-0.80)	<0.001
pLVAD	0.59 (0.55-0.62)	<0.001	0.63 (0.59-0.67)	<0.001	0.68 (0.63-0.72)	<0.001
ECMO	0.92 (0.86-0.99)	0.026	1.02 (0.94-1.10)	0.620	0.94 (0.87-1.02)	0.119
RHC	0.74 (0.72-0.77)	<0.001	0.77 (0.75-0.80)	<0.001	0.82 (0.79-0.85)	<0.001
Advanced Heart Failure Therapy	0.52 (0.48-0.55)	<0.001	0.55 (0.51-0.59)	<0.001	0.57 (0.53-0.61)	<0.001
LVAD	0.47 (0.43-0.51)	<0.001	0.49 (0.45-0.54)	<0.001	0.51 (0.46-0.56)	<0.001
Heart Transplant	0.62 (0.56-0.68)	<0.001	0.68 (0.61-0.75)	<0.001	0.70 (0.63-0.78)	<0.001
**Clinical Outcomes**						
In-hospital Mortality	1.26 (1.23-1.30)	<0.001	1.22 (1.19-1.26)	<0.001	1.20 (1.17-1.23)	<0.001
IMV	1.19 (1.16-1.22)	<0.001	1.20 (1.17-1.23)	<0.001	1.16 (1.13-1.20)	<0.001
Major Bleeding	0.95 (0.92-0.98)	<0.001	0.99 (0.96-1.02)	0.685	0.96 (0.93-0.98)	0.005
AKI	0.76 (0.74-0.78)	<0.001	0.75 (0.73-0.76)	<0.001	0.75 (0.73-0.78)	<0.001
AKI Requiring Dialysis	0.89 (0.86-0.93)	<0.001	0.92 (0.88-0.96)	<0.001	0.93 (0.89-0.98)	0.004
Stroke	1.69 (1.39-2.06)	<0.001	1.78 (1.44-2.19)	<0.001	1.73 (1.40-2.13)	<0.001

Procedural outcomes include interventions performed at any point during hospitalization. All analyses involve multivariate logistic regression and are survey weight adjusted. Model 1 includes adjustment with year of NIS data, age group, race, household income quartile, insurance status, hospital type and teaching status. Model 2 includes adjustment with all model 1 covariates as well as past medical history of diabetes mellitus, hypertension, dyslipidemia, tobacco use, obesity, chronic kidney disease, liver disease, peripheral vascular disease, coronary artery disease, chronic heart failure, valvular heart disease, stroke, prior PCI, and prior CABG.
Abbreviations: AMI-CS, acute myocardial infarction cardiogenic shock; MCS, mechanical support device; RHC, right heart catheterization; IMV, invasive mechanical ventilation; AKI, acute kidney injury.

Supplementary Table S4. Adjusted odds ratios of procedural utilization during AMI-CS and Non-AMI-CS hospitalizations in female patients by race.
*p<0.05

Procedure	Black	Hispanic	Asian & Pacific Islander	Native American
**AMI-CS**				
PCI	0.79 (0.73-0.87)*	0.91 (0.82-1.00)*	0.97 (0.84-1.11)	0.90 (0.65-1.26)
CABG	0.82 (0.73-0.92)*	1.00 (0.87-1.15)	0.88 (0.71-1.10)	1.62 (1.10-2.39)*
IABP	0.88 (0.80-0.97)*`	1.06 (0.95-1.18)	1.10 (0.93-1.29)	1.31 (0.93-1.87)
PLVAD	0.87 (0.76-0.99)*	0.89 (0.76-1.03)	0.97 (0.76-1.22)	1.24 (0.78-1.98)
ECMO	0.69 (0.51-0.93)*	0.85 (0.60-1.19)	1.12 (0.71-1.76)	0.93 (0.33-2.64)
RHC	1.07 (0.97-1.19)	1.05 (0.93-1.19)	1.02 (0.86-1.21)	1.04 (0.71-1.52)
**Non-AMI-CS**				
PCI	0.51 (0.40-0.63)*	0.75 (0.58-0.98)*	0.74 (0.47-1.16)	0.80 (0.29-2.21)
CABG	0.58 (0.50-0.67)*	0.95 (0.80-1.12)	1.31 (1.02-1.68)*	1.32 (0.73-2.40)
IABP	0.94 (0.83-1.06)	1.00 (0.85-1.19)	1.16 (0.90-1.49)	1.03 (0.57-1.87)
PLVAD	1.05 (0.92-1.20)	0.76 (0.61-0.95)*	1.02 (0.73-1.41)	1.07 (0.55-2.09)
ECMO	0.93 (0.80-1.09)	1.06 (0.85-1.31)	1.09 (0.78-1.52)	0.76 (0.36-1.62)
RHC	1.27 (1.18-1.36)*	0.97 (0.87-1.09)	0.88 (0.75-1.03)	0.69 (0.47-1.03)

White female patients were used as the reference group. Procedural outcomes include interventions performed at any point during hospitalization. All analyses involve multivariate logistic regression and are survey weight adjusted. Model includes adjustment with year of NIS data, age group, race, household income quartile, insurance status, hospital type and teaching status, diabetes mellitus, hypertension, dyslipidemia, tobacco use, obesity, chronic kidney disease, liver disease, peripheral vascular disease, coronary artery disease, chronic heart failure, valvular heart disease, stroke, prior PCI, and prior CABG.
Abbreviations: AMI-CS, acute myocardial infarction cardiogenic shock; MCS, mechanical support device; RHC, right heart catheterization; IMV, invasive mechanical ventilation; AKI, acute kidney injury.
